# Management of infected radicular cyst by surgical decompression

**DOI:** 10.4103/0972-0707.71651

**Published:** 2010

**Authors:** S Balaji Tandri

**Affiliations:** Department of Conservative Dentistry and Endodontics, Narayana Dental College and Hospital, India

**Keywords:** Calcium hydroxide, decompression, radicular cyst

## Abstract

Treatment of maxillary incisor with an associated cystic lesion by conventional endodontic therapy combined with decompression is reported. Although small cystic lesions frequently heal simply with endodontic therapy, larger lesions may need additional treatment. In some cases, when surgical enucleation is elected, there is a chance for inadvertent injury to adjacent teeth or structures even though periapical surgery has a role in endodontics. Therefore a more conservative approach of decompression and a workable protocol for this was attempted with success and is presented here.

## INTRODUCTION

Conventional non surgical root canal therapy is the treatment of choice in management of teeth with large periapical lesions.[1] When this treatment fails in resolving the periradicular pathosis alternative strategies like nonsurgical retreatment or periapical surgery must be considered. In case of very extensive lesions, as there is a chance for inadvertent undesirable consequences when surgical curettage is done, the procedures like Marsupialization or tube decompression are indicated.[2] Marsupialization is a surgical decompression procedure used to reduce large periapical lesions without periapical curettage.[3] Decompression allows continuous drainage from periapical lesion, which eliminates conditions favoring expansion of periapical pathosis resulting in healing by osseous regeneration.[4] Hence this paper is to discuss conventional management of large periapical pathosis without surgical curettage.

## CASE REPORT

**Figure 1:**
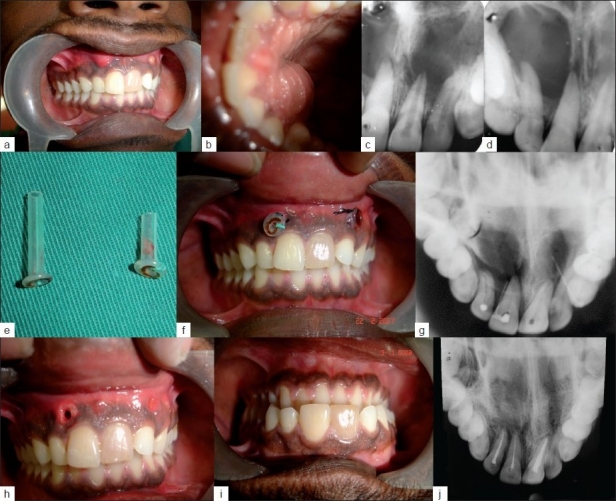
a) Picture showing swelling in labial vestibule region, b) Picture showing swelling in palatal mucosa, c) Radiograph of 11, 12 regions which reveal radiolucency involving periapical region, d) Radiograph of 21, 22 regions which reveal radiolucency involving periapical region, e) Picture of the cannula, f) Insertion and stabilization of cannula by interrupted sutures, g) Occlusal radiograph confirming the position of cannula, h) Picture of the clinical appearance of the channel, i) Picture of the healed aperture, j) Occlusal radiograph of 11, 12 and 21 region showing trabecular pattern throughout lesion after 1year

A 21 year old male Patient came to Dental office with a chief complaint of swelling in the upper front tooth region both front and behind the teeth for the past 6 months. Patient had history of childhood accident. He revealed that when the pus gets collected the swelling increases in size and then discharges through palatal aspect. Once the pus is discharged the swelling subsides and then recurs after some days. The intra-oral examination revealed two swellings in the labial vestibule in relation to 11, 12 and 21, 22 regions and size of 2×2 cms and 3×2cms respectively with discoloration of 11 [Figure 1a]. Swelling is also seen in the palatal region extending from 13 to 23 regions and is of size 4×3 cms [Figure 1b]. The swellings were soft and fluctuant in nature. Electric and thermal pulp vitality testing showed negative response in 11, 12 and 21 while adjacent teeth showed normal response. Teeth were painless to vertical percussion. Initially an IOPA was taken to know the extent of lesion which revealed a lesion involving periapical region of 11, 12 and 21, 22 regions respectively [Figures 1c and 1d]. The extent of periapical radiolucency was 3×4 cms and 4.5×5cms. A fine needle aspiration of the swelling revealed a discharge containing pus and blood. A presumptive diagnosis of infected radicular cyst in 11, 12 and 21 was made. Root canal treatment of 11, 12, 21 and surgical decompression in periapical region was planned.

Lignocaine with 2% Adrenaline was administered. Access opening, pulp extirpation, working length determination, cleaning and shaping was done in 11, 12 and 21. Calcium hydroxide intracanal medicament was given for a week and then obturated.

A 1.5cms vertical incision was made with BP blade no 15 at the mucoperiosteum between root eminences of 11, 12 and 22, 23 respectively, the surgical site was irrigated with saline and deep curettage was done to remove granulation tissues. A radiopaque flanged cannula of length 2.5 cms on right quadrant and 4cms length on left quadrant were inserted into the depth of the cavity [Figure 1e]. Two interrupted sutures were placed to stabilize the drain on either side [Figure 1f].

A Radiograph was taken to confirm the position of the cannula. The patient was recalled after 48 hours for irrigation with normal saline and sutures removed, adjustment of cannula for tissue comfort was done [Figures 1g and 1h]. The patient was instructed to perform self irrigation of the lesion with normal saline using plastic syringe and needle after removing the cannula. The patient was instructed to contact the dentist immediately if the cannula cannot be reinserted. Patient was recalled every fortnight to monitor the length of cannula.

After 7 weeks the drain was removed with instruction to continue irrigating the aperture. Two weeks later healing was nearly complete [Figure 1i]. The patient was recalled after 1 year and occlusal radiograph was taken to evaluate periradicular healing [Figure 1j].

## DISCUSSION

The treatment options for large periapical lesions ranges from conventional nonsurgical root canal treatment with long term calcium hydroxide therapy to various surgical interventions. Leaving the access point open for continued drainage is not a new procedure, and this technique has often been used for cases in which the endodontic lesions are large and the exudates from the root canal cannot be controlled.[3,5–7]

Decompression procedure reduces the size of the lesion so that surgical intervention is unnecessary or if necessary will be limited to the immediate periradicular tissues of involved teeth. The procedure disrupts the integrity of lesion wall, eliminates internal osmotic pressure differential and promotes healing by osseous regeneration.[7–9]

The internal cavity of cystic lesions is filled with cellular debris, serum albumin and high molecular weight protein degradation products, creating higher osmotic pressure inside the cavity than surrounding tissues.[10,11] The well developed walls of the lesion composed primarily dense collagen fibers acting like semi permeable membrane which does not allow osmotic equilibrium. As these lesions have an internal pressure gradient greater than surrounding tissues fluid is attracted into the cavity by osmotic pressure and expansion of lesion continues as long as the walls of the lesion is intact.[12,13]

No clinical studies provided evidence relating the correct time to remove the cannula and allow channel to heal. Case reports indicate that the length of the time cannula was inserted for decompression varies from 5 weeks to 14 months.[5,7]

The following radiographic and clinical criteria are suggested for termination of decompression. The radiograph should reveal a thin delicate trabecular pattern throughout the radiolucent area and cavity should show clinical evidence of progressive reduction in size resulting need to reduce length of cannula several times. Finally there should be no untoward signs or symptoms of the lesion like purulent discharge, pain etc.[14]
